# Protein Biomarkers Shared by Multiple Neurodegenerative Diseases Are Calmodulin-Binding Proteins Offering Novel and Potentially Universal Therapeutic Targets

**DOI:** 10.3390/jcm12227045

**Published:** 2023-11-11

**Authors:** Danton H. O’Day

**Affiliations:** 1 Department of Biology, University of Toronto Mississauga, Mississauga, ON L5L 1C6, Canada; danton.oday@utoronto.ca; 2 Department of Cell and Systems Biology, University of Toronto, Toronto, ON M5S 3G5, Canada

**Keywords:** protein biomarkers, neurodegeneration, calmodulin-binding proteins, risk factor proteins, toxic protein aggregation, therapeutic targets, calmodulin hypothesis

## Abstract

Seven major neurodegenerative diseases and their variants share many overlapping biomarkers that are calmodulin-binding proteins: Alzheimer’s disease (AD), amyotrophic lateral sclerosis (ALS), frontotemporal lobar dementia (FTD), Huntington’s disease (HD), Lewy body disease (LBD), multiple sclerosis (MS), and Parkinson’s disease (PD). Calcium dysregulation is an early and persistent event in each of these diseases, with calmodulin serving as an initial and primary target of increased cytosolic calcium. Considering the central role of calcium dysregulation and its downstream impact on calcium signaling, calmodulin has gained interest as a major regulator of neurodegenerative events. Here, we show that calmodulin serves a critical role in neurodegenerative diseases via binding to and regulating an abundance of biomarkers, many of which are involved in multiple neurodegenerative diseases. Of special interest are the shared functions of calmodulin in the generation of protein biomarker aggregates in AD, HD, LBD, and PD, where calmodulin not only binds to amyloid beta, pTau, alpha-synuclein, and mutant huntingtin but also, via its regulation of transglutaminase 2, converts them into toxic protein aggregates. It is suggested that several calmodulin binding proteins could immediately serve as primary drug targets, while combinations of calmodulin binding proteins could provide simultaneous insight into the onset and progression of multiple neurodegenerative diseases.

## 1. Introduction

A biological marker or “biomarker” has been defined as “a characteristic that is objectively measured and evaluated as an indicator of normal biological processes, pathogenic processes, or pharmacologic responses to a therapeutic intervention” [1]. A biomarker is useful not only in the diagnosis and assessment of progress of a specific neurological disease but also for the development and evaluation of therapies [2]. In 2016, an FDA task force listed seven classes of biomarkers: (1) diagnostic biomarkers; (2) prognostic biomarkers; (3) predictive biomarkers; (4) response biomarkers; (5) monitoring biomarkers; (6) safety biomarkers; (7) risk biomarkers. Biomarkers range from cognitive test results (e.g., self-administered gerocognitive exam (SAGE); mini-mental state exam (MMSE)) to brain imaging (e.g., positron emission tomography (PET) scans; magnetic resonance imaging (MRI)) and analysis of biological fluids (cerebrospinal fluid (CSF); blood; saliva; urine) and tissue (e.g., skin). Some of them require expensive infrastructure (i.e., brain imaging) or are invasive (e.g., CSF), while others are less (e.g., blood) or non-invasive (e.g., urine) [2]. Regardless, each biomarker detection method has specific problems as exemplified by those issues facing blood-based assays. Numerous factors interfere with the use of blood for evaluating biomarkers, some of which may apply to other bodily fluids: albumin interferes with detection of low-abundance peptides; immune response removes biomarkers; biomarker aggregation; enzymatic degradation; clearance by chaperones [3]. The most accurate diagnostic methods for neurodegenerative diseases are invasive (e.g., CSF) or labor intensive, expensive, and not always accessible (e.g., MRI, PET), so the development of less or non-invasive, inexpensive, and precise biofluid (i.e., blood, urine, saliva) biomarkers is paramount. The focus here is on biological fluid protein biomarkers, some of which are risk and hallmark proteins, associated with seven major neurodegenerative diseases: Alzheimer’s disease (AD), amyotrophic lateral sclerosis (ALS), frontotemporal lobar dementia (FTD), Huntington’s disease (HD), Lewy body disease (LBD), multiple sclerosis (MS), and Parkinson’s disease (PD) [4,5,6,7,8]. Most of these diseases have crossover variants that share overlapping biomarkers. The major neurodegenerative diseases are temporally and pathophysiologically characterized by a combination of relatively constant attributes: calcium dysregulation; neuroinflammation; oxidative stress; age-linked occurrence; progressive neuron dysfunction/death; risk factors (genetics, gender, health, physical fitness, etc.). In addition, specific toxic proteins are the classic hallmarks of certain diseases: AD (amyloid beta, Aβ; phosphorylated Tau, pTau); HD (huntingtin, HTT); PD (α-synuclein, αSyn). Coupled with this are genetic mutations that either dictate or contribute to the disease, as discussed below.

Calcium signaling is essential for the basic function and survival of neurons [9,10]. An early, possibly initiating, event in most, if not all, neurodegenerative diseases is calcium dysregulation. The critical, uncontrolled increase in calcium levels and the disruption of downstream calcium signaling has been well studied in AD and HD [11,12,13,14]. In PD, calcium dysregulation is an early event and subsequent αSyn aggregation feeds back to augment calcium dyshomeostasis, further disrupting calcium signal transduction and CaM-mediated events [15,16,17]. Calcium dysregulation is a determining event underlying neurodegeneration and the specific loss of motor neurons in ALS [18,19]. The role of calcium dysregulation has also been reviewed for MS [20]. Calcium dysregulation is an early event that impacts vulnerable neurons in LBD and impacts downstream calcium-binding proteins in FTD [19,21].

The major initial downstream target of calcium dysregulation is calmodulin (CaM), a calcium-binding protein that can bind to and regulate hundreds of proteins. The structure, mode of action, and diverse and essential functions of this small (149aa; 16.7 kDa) and highly conserved protein are well known [22]. CaM has multiple, critical functions in neurons where it regulates learning and memory, calcium homeostasis, synaptic signaling, neurotransmitter release, and neuroplasticity, among many other events. Its function in AD and other neurodegenerative diseases has been analyzed [8,23]. CaM has long been known as an essential protein that has changed little over its evolution. CaM’s main function is to mediate calcium signal transduction events by binding to and regulating target proteins called CaM-binding proteins (CaMBPs). The complex binding interactions and the identification, classification, and validation of calmodulin-binding domains (CaMBDs) have been well reviewed [24,25,26,27]. CaM-dependent protein kinase II (CaMKII), protein phosphatase 2B (PP2B) (calcineurin), N-methyl-D-aspartate receptor (NMDAR), acetylcholine receptor (AchR), adenosine A2A receptor, and cyclin-dependent kinase 5 (cdk5) are all experimentally validated CaMBDs involved in a diverse array of neurodegenerative diseases and their roles have also been well reviewed [8,28,29]. For example, the classic CaMBPs CaMKII and PP2B are primary enzymes in models of learning and memory (long-term potentiation (LTP) and long-term depression (LTD) [30]. CaMKII has long been known to be involved in neuropsychiatric disorders including depression, epilepsy, and schizophrenia [31]. However, for this review, the focus is on disease-defining biomarkers for AD, ALS, FTD, HD, LBD, MS, and PD that are CaMBPs. Any CaMBP biomarkers discussed below that are linked to more than one neurodegenerative disease are listed in Table 1.

## 2. AD

Each year, around 55 million dementia cases exist worldwide of which 60–80% are due to AD, a progressive neurodegenerative disease for which there is no cure and few available options for symptom treatment [32,33]. Characterized by personality changes with a progressive loss of memory and cognition, AD begins ten or more years before symptoms are diagnosed, beginning with mild cognitive impairment (MCI) that may or may not progress to dementia. The presence of senile plaques rich in amyloid beta (Aβ), neurofibrillary tangles (NFTs) of pTau, and neurodegeneration in brain samples have historically defined the disease post mortem. In living patients, various combinations of biomarkers generated by cognitive tests, brain scans, and analysis of bodily fluids provide data for AD diagnosis [34]. Early-onset AD (familial AD) is driven by genetic mutations in specific genes (i.e., AβPP, presenilin 1 and 2 (PSEN1, PSEN2)) and typically begins before age 40. Late-onset AD (LOAD) is a multifactorial syndrome that typically begins after age 65. PSEN1 mutations have also been discovered in some atypical AD variants as well as in FTD, DLB, and PD [35]. While a number of risk genes are linked to LOAD (i.e., APOE4e4), age and a long list of other risk factors (e.g., gender, socioeconomic and educational status, diabetes) are associated with it [33]. Fernández-Calle et al. have reviewed in excellent detail the impact of APOE mutations in AD, ALS, MS, PD, LBD, and other neurodegenerative diseases [36]. APOE4 is recognized as a biomarker and risk factor for AD, LBD, and PD. For PD, it appears that APOE4 mutations cause a higher rate of cognitive dysfunction and chance of developing dementia, but they do not impact motor degeneration. While, for various reasons, APOE4 mutations are not recognized as a risk factor for ALS or MS, they can serve as biomarkers due to their impact on those diseases.

CaM has been shown to be central to both the onset and progression of AD [7,29]. CaM is involved in the initiation and progression of amyloidogenesis. AβPP, the source of Aβ, binds to CaM an event that determines processing via the amyloidogenic vs. non-amyloidogenic pathways [37]. The first enzyme in amyloidogenesis is BACE1, which not only binds to CaM but is activated two-fold by this binding [38]. After this, a-secretase completes the process, releasing Aβ. a-Secretase comprises four subunits, one of which is PSEN, an experimentally verified CaMBP [39]. The subsequent aggregation of Aβ monomers, which individually bind to CaM, is enhanced by CaM binding [40]. Monomeric Aβ has multiple normal physiological functions and is non-toxic but becomes toxic upon oligomerization, with oligomers playing a key role in AD pathogenesis [41,42]. Thus, the oligomerization of Aβ driven by CaM binding could be a primary cause of neurodegeneration in AD and thus a viable therapeutic target. As the disease progresses, CaM continues to be involved [7]. 

The second classic biomarker for AD is neurofibrillary tangles (NFTs), which are primarily accumulations of modified Tau proteins. Tau is a multifunctional, microtubule-associated protein family member, which upon phosphorylation (pTau) is dislodged from microtubules where continued phosphorylation on multiple sites, which occurs in AD, can generate toxic forms of the protein [43]. Soluble non-toxic pTau monomers become toxic when they oligomerize at an early stage in the formation of neurofibrillary tangles (NFTs). Calmodulin binds to Tau’s tubulin-binding site, and this binding may be involved in microtubule stability, an event linked to neurodegeneration [44]. However, unlike the situation with Aβ, the potential involvement of CaM in Tau oligomerization appears to remain unstudied. Transglutamase 2 (TGM2) is associated with multiple neurodegenerative diseases and expressed at elevated levels in AD brains where it localizes to senile plaques and NFTs [45]. TGM2 catalyzes the cross-linking of AβPP, Aβ, and tau, contributing to Aβ oligomers and paired Tau helical filaments (PHFs) [46,47]. TGM2 is a CaMBP that is also involved in ALS, HD, LBD, and PD, as discussed below [48]. 

Aβ and pTau continue to be the primary biomarkers for evaluating AD, and their levels in blood serum coupled with neurofilament light chain (NfL) or neurogranin (Ng, RCE, p17, BICKS), indicators of neuron damage, could be a way to track AD before symptoms appear [49,50]. While NfL is not a CaMBP, Ng is an experimentally validated CaMBP that is also released from degenerating synapses and neurons, and serves as a non-specific biomarker for AD as well as for LBD, HD, PD, and Creutzfeldt–Jakob disease, among other brain diseases [51,52]. Ng binds to calcium-free apoCaM, sequestering and localizing it to synapses where it plays a role in models of learning and memory: LTP and LTD [51]. The loss of synapses in AD is thus linked to memory malfunction due in part to the loss of Ng function [53].

Found in astrocytes, microglia, and neurons, ATP-binding cassette protein A1 (ABCA1) is a dominant ABC transporter in the brain and is central to cholesterol mobilization and Aβ clearance. Polymorphisms in ABCA1 have been linked to increased susceptibility to neurodegenerative diseases. In keeping with this, ABCA1 has reduced levels of expression and activity in AD, PD, LBD, MS, and traumatic brain injury (TBI) [54,55,56]. Calcium-dependent binding of CaM to ABCA1 (via a 1–5–8–14 motif) in vivo stabilizes it and prevents its digestion by calpain [57]. A major risk factors for late-onset AD, clusterin (CLU, apolipoprotein J) has been linked to the clearance of Aβ with contradictory results [58]. CLU, which has a single CaMBP, indicating that it binds to CaM, is also involved in MS and PD [8,59]. 

Continued research continues to identify potentially useful AD biomarkers that bind to CaM. Triggering receptor expressed on myeloid cells 2 (TREM2) has been linked to neuroinflammatory events in AD, ALS, FTD, and PD [28,60,61]. This presumptive CaMBP contains a single CaMBD that needs to be validated [8]. Levels of the multifunctional antimicrobial, immunoregulatory iron-binding glycopeptide lactoferrin (LF) decrease in the saliva of AD individuals but increase in the brain where LF is found around both senile plaques and NFTs [62,63]. Human LF shows a novel, non-canonical calcium-dependent binding to CaM that is involved in its immunoregulatory functions [64]. LF appears to provide neuroprotection since it prevents prion-mediated cell death, ameliorates cerebral hemorrhage, and localizes to undamaged brain regions in PD sufferers [63]. Levels of LF in brain lesions also implicate it in ALS as well as other neurodegenerative diseases (e.g., Pick’s, Prion diseases) where it is possible that it functions by improving immune function and modulating toxic reactive oxygen species (ROS) [62,63]. Based on this and other extensive data, salivary LF can serve as a biomarker for the diagnosis and monitoring of the progress of neurodegeneration [62]. 

AD is a heterogeneous disease with a diversity of subtypes. In addition to clinical variants that are reflected by deficits in language, progressive aphasia, visuospatial atrophy, and other subtypes, individual cases can express pathologies found in other neurodegenerative diseases including FTD, LBD, PD, and vascular dementia [65]. Thus, the CaMBP αSyn is presented in AD patients with PD symptomology. 

## 3. HD

A monogenic (*HTT* gene on chromosome 4) neurodegenerative disease, Huntington’s disease (HD) is detectable via gene testing prior to symptom appearance [66]. Characteristic age-dependent symptoms include uncontrollable muscle movements, cognitive impairment, and psychological issues due to neuronal damage in the striatum and cortex [66,67]. Despite the availability of early detection, there is no cure or disease-modifying therapy for HD. The *HTT* gene encodes the ubiquitous huntingtin (HTT) protein with an extensive polyglutamine repeat that when mutated (mHTT) possesses a more extensive and toxic polyglutamine repeat that is prone to aggregation [68]. mHTT is a blood biomarker for HD that oligomerizes to form fibrils and inclusions in neurons. HTT and mHTT are experimentally proven calcium-dependent CaMBPs, with CaM binding increasing with the length of the polyQ repeat [69]. CaM and HTT also colocalize with transglutaminase (TGM2) an enzyme that not only binds to and is activated by CaM but also hydrolyzes the poly-Q repeat [48,70,71]. TGM2 also cross-links HTT protein fragments to form insoluble deposits, an event that is enhanced by binding of CaM to the enzyme [45]. In further support of the importance of TGM2 in HD is evidence that the level of transglutaminase activity is increased in HD brain tissue, especially in the superior frontal cortex [72]. Targeting the CaM binding of TGM2 to increase its activity could generate shorter polyQ sequences in HD patients and lower the risk of protein aggregation.

Other protein biomarkers for HD that are also linked to other neurodegenerative diseases are NfL, CLU, TDP-43, and Tau [67,68]. As discussed above, Tau is a CaMBP and CLU possesses a CaMBD, while the others (NfL, TDP-43) do not bind to CaM. Despite their lack of specificity to HD, NfL, TDP-43, and Tau levels signify the extent of neuronal degeneration and thus serve as strong collaborative biomarkers for disease onset and progression for HD as well as other neurodegenerative diseases. The CaMBP biomarker αSyn is also linked to HD since it forms separate aggregates within HTT inclusions in a mouse model for HD, where it counteracts polyQ toxicity [73]. A few dozen unvalidated biomarkers from unreplicated studies have also been identified but await additional support [67]. 

## 4. PD

A multifactorial disease with variable rates of progression, PD is caused by a complex combination of genetic, personal (e.g., diet, ethnicity, gender, lifestyle), and environmental factors (e.g., chemical and pesticide exposure) [74,75]. The disease affects ~1% of individuals aged 60 years or older. It is primarily a motor disease that becomes evident when 70–80% of nigrostriatal dopamine neurons have degenerated, preventing effective therapeutic intercession. Prior to this, other non-motor neurodegenerative changes underlie behavioral changes, such as loss of motivation, providing a window for therapeutic intervention, although these changes are not PD specific. A small percentage (5–10%) of PD cases are due to genetic mutations in αSyn and LRRK2 (autosomal-dominant) or Parkin, PINK1 and DJ-1 (autosomal-recessive), or to GBA, the most common genetic risk factor [76]. A number of investigators have identified potentially useful biomarkers for PD, of which several are proven or putative CaMBPs, as discussed below: αSyn, LRRK2, Park7, PINK1, PRKN, DJ1, APOE4, and GBA [8,75,77]. CaM also binds to and inhibits the D2-dopamine receptor (D2DR) [78]. A polymorphism of the CaMBP beta-secretase (BACE1) is also linked to PD [79]. 

The need to identify precise PD-specific biomarkers is critical. However, a problem exists: PD is a complex of disease subtypes with multiple pathologies [77,80,81,82]. Due to the toxic aggregation of αSyn and its transformation into Lewy bodies (LBs), a major pathological attribute of PD, the disease is part of the family of synucleinopathies that includes LBD and others. LBs, a pathological feature of hereditary and sporadic PD, are primarily aggregates of misfolded αSyn that, depending on the subtype, are associated with other experimentally validated CaMBPs (Aβ, Tau) and others with identified CaMBDs (GBA, TDP-43, LRRK2, PINK1) [8]. LRRK2, TDP-43, and two isoforms of PINK1 each have a single valid calcium-dependent CaMBD. With two canonical calcium-binding CaMBDs and one IQ motif, GBA is a strong contender as a functional CaMBP. Each of these protein biomarkers is linked to various disease attributes. For example, the concomitant presence of AD and LB pathology is a stronger predictor of subsequent cognitive decline and dementia in PD than individual factors [83]. These pathologies can act synergistically, as shown by the induction of αSyn phosphorylation at Ser129 by Aβ [84]. The phosphorylation of Ser129 is correlated with disease severity and αSyn aggregation [85]. TGM2 not only phosphorylates Ser129 but also localizes within LBs. Prior to this, in PD and LBD, it catalyzes the cross-linking of αSyn to generate insoluble aggregates as components of LB [45]. Coupled with this, PINK1, a protein with a CaMBD, phosphorylates TGM2, increasing its protein stability, which in turn could increase αSyn aggregation. Calcium-dependent CaM binding enhances αSyn fibril formation [86,87]. 

In addition, coupled with LBs, other pathologies can be present in PD brains including Aβ senile plaques and neurofibrillary tangles (NFTs). The CaMBPs Aβ, tau, and αSyn are all found in LBs of PD and LBD [17,88]. To add to this complexity, Aβ and pTau, the precursors of plaques and tangles, interact with αSyn, enhancing cognitive decline [89]. Since Aβ, Tau, and αSyn can all individually bind to CaM, the potential regulatory complexities and their implications in neurodegeneration are enormous yet remain unstudied. They may also offer new therapeutic routes. Recently, the concept of combining biomarkers has been suggested, which would allow more precise evaluation of PD [75]. The authors of that study found that serum NfL levels and APOE and GBA status combined with “previously validated clinical measures can provide a better prediction of several aspects of PD progression” than clinical measures. As discussed, APOE is a proven CaMBP and GBA is a putative one, with two canonical calcium-binding CaMBDs plus a single calcium-independent binding IQ motif [23].

## 5. Lewy Body Diseases

LBD (or dementia with Lewy bodies, DLB), which typically begins after age 50, represents the second most common neurodegenerative disease (~20% of dementia cases). It is characterized by sleep disorders and early cognitive decline, especially changes in alertness and attention, followed by hallucinations and tremors, among other symptoms close to those seen in AD [90]. Individuals with LBD typically also have AD and other pathologies associated with plasma Aβ and pTau. On the other hand, other CaMBP biomarkers for AD (e.g., Ng) are not associated with DLB [91]. A biosymposium report on LBD biomarkers from bodily fluids revealed the state of the art for this subject but did not provide a consensus on the best biomarkers for evaluating the onset or progression of the disease and its symptoms [92]. While their ability to bind CaM was not reviewed, details about research into analyses involving αSyn, Aβ, tau, APOE, TDP-43, and NfL led to considerations about what needs to be achieved to improve biomarkers and diagnostic criteria for LBD with and without AD pathology. In addition to those biomarkers, several other LBD biomarkers that are CaMBPs or possess CaMBDs have been recognized: BIN1, GBA, and TMEM175 [8,93]. 

LBs, a pathological feature of LBD and hereditary and sporadic PD, are aggregates of misfolded αSyn that, depending on the disease, are associated with other proteins: Aβ, GBA, TDP-43, LRRK2, PINK1, Parkin, Tau. These protein associates are linked to various disease attributes. For example, the concomitant presence of AD and LB pathology is a stronger predictor of PD subsequent cognitive decline and dementia than individual factors [83]. These pathologies can act synergistically as shown by the induction of αSyn phosphorylation at Ser129 by Aβ, as discussed above for PD, which is correlated with αSyn aggregation and disease severity [84,85]. 

## 6. FTD

The third most common progressive neurodegenerative disease (~20% of dementia cases), FTD is a heterogeneous disease, originating in the frontal and/or temporal lobes, that affects behavior, language, and executive function, beginning prior to age 65 [94,95]. Since FTD symptoms are common to several primary psychiatric disorders including autism spectrum disorders, bipolar disorder, and schizophrenia, family history, neurologic testing, neuroimaging, and genetic testing can be used to rule other diseases out. In addition, FTD variants exist that share AD, ALS and PD neuropathology. Three genes (chromosome 9 open reading frame 72 (C9orf72), granulin (GRN) and Tau) make up ~30–50% of familial FTD [95]. Around 5% of FTD cases are a result of Tau gene (MAPT) mutations [96]. A dozen or so less common (5% of FTD mutations) mutations have also been identified [97,98]. While ABCA7 is involved in monogenic FTD, genetic analyses have revealed that pathogenic variants in ABCA1, C9orf72, CHCHD10, CTSF, DCTN1, FUS, GRN, OPTN, Parkin, PSEN1, SQSTM1, TARDBP, TBK1, Tau, and VCP were found in all FTD clinical subtypes [99,100]. Half of these variants are either proven CaMBPs (i.e., ABCA7, PSEN1, Tau) or proteins containing classic calcium-dependent CaMBDs (i.e., ABCA1, C9orf72, FUS, SQSTM1, TBK1) many with links to other neurodegenerative diseases (Table 1). 

Clearly, a large number of genetic biomarkers have been linked to FTD, but now, the issue is to define those that can differentiate between the different FTD variants [95,101]. The presence of CaMBP biomarkers for other diseases (e.g., AD: Aβ, pTau; PD: αSyn) can differentiate some, but other disease variants are less easy to determine. For example, not only do FTD and ALS share overlapping biomarkers, including the presumptive CaMBP TDP-43, but approximately 15% of FTD cases also share ALS-like motor symptoms, while ALS sufferers share a similar percentage of FTD symptomology [94]. Mutations in the putative CaMBP TREM2 increase the risk for familial FTD but not LBD [60]. AD symptomology coupled with age-dependent accumulation of Aβ has also been verified in a significant proportion of FTD patients [102]. A sporadic neurodegenerative disease, multiple system atrophy (MSA) is also considered to be a subtype of FTD associated with αSyn deposits [103]. 

## 7. ALS

Typically occurring around age 60 and predominantly in males, amyotrophic lateral sclerosis (ALS; Lou Gehrig’s disease) is a progressive neurodegenerative disease impacting motor neurons in the brain and spinal cord that control voluntary muscles [19,104]. As these neurons degenerate and die, the muscles they control also degenerate and atrophy, affecting limb strength, talking, and swallowing, among other functions, eventually leading to death within 3–5 years from disease onset. While around 70% of cases affect both upper and lower motor neurons, less often, either upper or lower motor neurons can be affected separately, thus defining ALS subclasses. Clinically heterogeneous, there are no specific biomarkers for amyotrophic lateral sclerosis (ALS) and only 10–15% of cases have any genetic basis for this incurable disease [18]. Most cases are sporadic, with around 5–10% of cases caused by mutations in a variety of genes. Of these, 25–40% of genetic ALS is due to a defect in the C9orf72 gene, while 12–20% is caused by mutations in superoxide dismutase 1 (SOD1). This year, the FDA approved a SOD1 antisense oligonucleotide (Qalsody (tofersen)) to reduce levels of the enzyme in ALS patients. Like other neurodegenerative diseases, calcium dysregulation involving glutamate receptors is an early event in ALS, leading primarily to excitotoxicity due to the malfunction of mitochondria and endoplasmic reticulum. Linked to calcium dysregulation, several CaMBP risk and marker proteins overlap between FTD and ALS: TDP-43, SQSTM1, FUS, TBK1, and C9orf72 [105]. Of these, TDP-43 is considered to be a strong hallmark for ALS. In addition, several other proven and putative CaMBPs involved in other neurodegenerative diseases have also been linked to ALS including APOE, TREM2, and PP2B [8]. The CaMBP TGM2 has been shown to accelerate neuroinflammation in ALS [106]. In addition, the experimentally validated CaMBPs AβPP and Aβ localize to spinal motor neurons in individuals with ALS [107,108]. αSyn-positive LBs, containing SOD1 and TDP43, have been detected in ALS patients [109]. Many clinical trials are underway in the U.S. and Canada with the majority focusing on therapies involving well-studied CaMBP biomarkers such as FUS, TDP-43, and SOD1, with some developing AI approaches to biomarker evaluation.

## 8. MS

A progressive, debilitating autoimmune disease with heterogeneous histopathological and radiological attributes, multiple sclerosis (MS) attacks the protective myelin sheath surrounding axons [20,55]. While MRI bioimaging is currently the most accurate way to reveal the disease and its status, the search for less expensive and more accessible biomarkers is underway, but few have been revealed [110]. Currently immunoglobulin levels in blood and serum provide an IgG index that is a useful MS biomarker. Because the diagnosis of ALS typically comes late in the disease, finding rapid and inexpensive biomarkers for its early detection is critical to finding a cure or ways to slow disease progression [111]. In the meantime, the utility of certain CaMBP biomarkers common to other neurodegenerative diseases has been studied as an evaluator of ALS status and progression including ABCA1, APOE, C9Orf72, FUS, and pTau/Tau [3,55,94,111,112,113] A recent study presents evidence that the CaMBP αSyn is a biomarker that could be used in the diagnosis and prediction of MS [114]. 

## 9. Discussion

The link between biomarkers involved in neurodegenerative diseases that are either proven or presumptive CaMBPs is extensive, with most of them involved in multiple diseases and/or their variants (Table 1). The classification of neurological diseases has been evolving as new research details are revealed, often debunking the concept of a typical neurodegenerative disease and, instead, supporting the use of neurodegenerative syndrome. A recent review of Lewy body diseases makes it clear that “typical” PD and other LB diseases do not exist, since the authors present detailed evidence that “PD, PDD, and LBD represent closely related but different, heterogeneous subtypes of an αSyn-associated disease spectrum” [82]. This idea can be extended to other neurodegenerative diseases where hallmarks, biomarkers, risk genes, and symptomology vary so widely between patients, diverse attempts to subclassify them only open new areas of uniqueness. For these and other reasons, researchers have begun referring to many neurodegenerative diseases as syndromes (e.g., Alzheimer’s syndrome, Parkinson’s syndrome). Furthermore, many diseases show combined pathologies (e.g., PD/AD pathology). Historically, most neurodegenerative diseases are classified based on late-stage hallmarks (e.g., plaques, NFTs, LBs) and their components that are evaluated after the symptoms of the disease have ravaged the sufferer’s mind and body. Finding early neurological biomarkers is critical. Here, we have shown that, coupled with one of the earliest events common to most, if not all, neurodegenerative diseases, calcium dysregulation is involved in regulating CaM that, in turn, binds to a diverse number of early biomarkers that are thematic for neurodegenerative diseases.

Finding a group of biomarker proteins that are expressed at different levels in different neurodegenerative diseases might provide a way to simultaneously assess the type of disease/syndrome using a single platform. For example, coupling disease-specific AD biomarker levels of Tau, pTau, and Aβ with non-specific biomarkers of neuronal degeneration and death (e.g., Ng) could provide more insight into disease progression. On the other hand, combining disease-specific biomarkers for one disease with those associated with other neurodegenerative diseases could yield insight into disease variants and their combinatorial symptoms [115].

**Figure 1:**
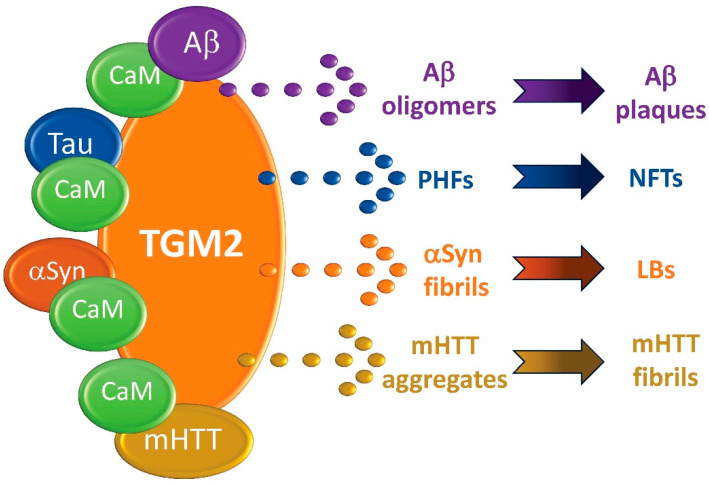
A common pathway for toxic protein aggregation in neurodegeneration.

As discussed above, calmodulin and its binding proteins play a major role as biomarkers of neurodegenerative diseases, but details about how they function in neurodegenerative events have only been revealed for a few key events. Likely the best example of this for all neurodegenerative diseases are the functions of CaMKII and PP2B in LTP and LTD, events that are impeded by calcium dysregulation [30]. But other examples are coming to light, one of which one is extremely significant. As summarized in Figure 1, the role of CaM in toxic protein aggregation in AD, HD, LBD, and PD is especially intriguing since it points to a common sequence of events that could lead to universal therapeutic targeting of neurodegenerative events. As discussed above and detailed elsewhere, CaM binding to Aβ drives oligomerization, producing toxic Aβ oligomers that in turn feed back to increase Aβ production [116]. CaM not only binds to but also has multiple functions in the aggregation of the biomarkers HTT, αSyn, and Tau, but the importance of the CaMBP TGM2 stands out because all four of the major toxic proteins (Aβ, αSyn, mHTT, Tau) are substrates for it [45]. In AD, TGM2 can cross-link Aβ to generate toxic oligomers as a precursor to the formation of senile plaques. By cross-linking Tau, TGM2 also functions in the formation of NFTs. In HD, TGM2 not only hydrolyzes mHTT but also cross-links the resultant mHTT fragments, forming insoluble aggregates an event increased by CaM activation of the enzyme. Similarly, in PD and LBD, TGM2 cross-links αSyn, causing the formation of insoluble aggregates as a precursor to LB formation. Preventing proteins from forming toxic aggregates/oligomers is just one area where targeting CaM and its CaMBPs could be useful, and specifically targeting the CaMBP TGM2 offers a therapeutic route for multiple neurodegenerative diseases. Since the enzyme and the biomarker substrates are all experimentally validated CaMBPs, the potential of finding a universal therapy for treating toxic peptide aggregation seems possible. 

This work adds further support for the Calmodulin hypothesis in neurodegeneration since numerous useful CaMBP crossover biomarkers and their critical functions in neurodegenerative diseases have been revealed [7,23,29]. The question now is how to use them effectively. CaM and its binding proteins lie at the heart of multiple neurodegenerative diseases; with the availability of multiple drugs and novel approaches, the way is open to develop CaM-based therapies. A long list of antagonists/inhibitors for CaM and specific CaMBPs exist that researchers can use immediately. CaM antagonists, which could be repurposed, have been used successfully to treat pancreatic cancer and the cancer-dependent event angiogenesis [117]. As another example, treatment of organ transplant patients with the CN inhibitor FK506 (tacrolimus) not only prevented organ rejection but led to a significantly lower incidence of dementia [118]. In keeping with this, treatment of a mouse model for AD with FK506 improved both neuronal morphology and object recognition [119]. FK506 is currently being investigated for use as a therapeutic treatment for neurodegeneration (ClinicalTrials.gov; accessed on 25 May 2023). In addition, new approaches in targeting CaMBPs are being developed: antisense oligonucleotides, miRNAs, and small interfering RNA [120]. Another way to further improve accuracy is to use machine learning and artificial intelligence to analyze combined results from multiple sources [34]. As a result, developing a panel of CaMBP biomarkers that could screen for the existence and progression of multiple neurodegenerative diseases is becoming a viable route.

## Figures and Tables

**Table 1:** Calmodulin Binding Protein Neurodegeneration Biomarkers.

**I. Experimentally Validated CaMBP Biomarkers**							
***Biomarker***	***AD***	***ALS***	***FTD***	***HD***	***LBD***	***MS***	***PD***
αSyn	✓*	✓	✓*	✓	✓	✓	✓
Aβ	✓	✓	✓*	-	✓*	✓	✓
ABCA1	✓	-	✓	-	✓	✓	✓
AβPP	✓	✓	-	-	-	-	-
APOE4	✓	✓	✓	-	✓	✓	✓
BACE1	✓	-	-	-	-	-	✓
HTT	-	-	-	✓	-	-	✓
LF	✓	✓	-	-	-	-	✓
Ng	✓	-	-	✓	✓	-	✓
PSEN1	✓	-	✓	-	✓	-	✓
Tau	✓	✓	✓	✓	✓	✓	✓
TGM2	✓	✓	-	✓	✓	-	✓
**II. Biomarkers with CaM-Binding Domains**							
***Biomarker***	***AD***	***ALS***	***FTD***	***HD***	***LBD***	***MS***	***PD***
ABCA7	✓	-	✓	-	-	-	✓
BIN1	✓	-	-	-	✓	-	-
C9orf72	-	✓	✓	-	-	-	-
CLU	✓	-	-	-	-	-	✓
FUS	-	✓	✓	-	-	-	-
GBA	-	✓	✓	-	✓	-	✓
LRRK2	-	-	-	-	✓	-	✓
PARK7	-	-	-	-	-	-	✓
PINK1	-	-	-	-	✓	-	✓
SQSTM1	✓*	✓	✓	-	-	-	-
TBK1	-	✓	✓	-	-	-	-
TDP-43	-	✓	✓	✓	✓	-	✓
TMEM175	-	✓	✓	-	-	-	✓
TREM2	✓	✓	✓	-	-	-	✓

*Legend*: The full names of the biomarkers are listed in the text; -, not determined; ✓, present; ✓*, present in some disease variants.

## Data Availability

Not applicable.

## Abbreviations

ABCA1	ATP-binding cassette protein A1
AchR	acetylcholine receptor
AD	Alzheimer’s disease
ALS	amyotrophic lateral sclerosis
APOE	apolipoprotein E
αSyn	alpha-synuclein
BIN1	bridging integrator 1
C9orf72	chromosome 9 open reading frame 72
CaM	calmodulin
CaMBD	calmodulin-binding domain
CaMBP	calmodulin-binding protein
CaMKII	calmodulin-dependent protein kinase II
cdk5	cyclin-dependent kinase 5
CH3L1/YKL-40	chitinase-3-like protein I
CHCHD10	coiled-coil–helix-coiled-coil–helix domain-containing protein 10
CLU	clusterin
CR1	complement receptor type 1
D2DR	D2-dopamine receptor
FTD	frontotemporal dementia
FUS	fused in sarcoma-RNA-binding protein
GBA	glucocerebrosidase
GRN	progranulin
HD	Huntington’s disease
LB	Lewy body
LBD	Lewy body disease
LF	lactoferrin
LRRK2	leucine-rich repeat kinase 2
MS	multiple sclerosis
NFTs	neurofibrillary tangles
Ng	neurogranin
NfL	neurofilament light chain
NMDAR	N-methyl-D-aspartate receptor
PD	Parkinson’s disease
PARK7	Parkinsonism-associated deglycase 7
PILRA	paired immunoglobin-like type 2 receptor alpha
PINK1	PTEN induced kinase 1
PP2B	calcineurin
ROS	reactive oxygen species
SNCA	α-synuclein
SQSTM1	sequestosome 1
TBI	traumatic brain injury
TGM1/2	transglutamase ½
TMEM175	transmembrane protein 175
TDP-43/TARDBP	TAR DNA-binding protein
TREM2	triggering receptor expressed on myeloid cells 2
VCP	valosin-containing protein
